# Pesticide contamination of lactating mothers’ milk in Latin America: a systematic review

**DOI:** 10.11606/s1518-8787.2024058005446

**Published:** 2024-04-25

**Authors:** Thalyta Mota Figueiredo, Jerusa da Mota Santana, Fernando Henrique Basilio Granzotto, Bianca Sampaio dos Anjos, Danilo Guerra, Laylla Mirella Galvão Azevedo, Marcos Pereira

**Affiliations:** I Universidade Federal do Recôncavo da Bahia Centro de Ciências da Saúde Santo Antônio de Jesus BA Brasil Universidade Federal do Recôncavo da Bahia. Centro de Ciências da Saúde. Santo Antônio de Jesus, BA, Brasil; II Universidade Federal do Oeste da Bahia Centro das Ciências Biológicas e da Saúde Barreiras BA Brasil Universidade Federal do Oeste da Bahia. Centro das Ciências Biológicas e da Saúde. Barreiras, BA, Brasil; III Universidade Federal da Bahia Instituto de Saúde Coletiva Programa de Pós-Graduação em Saúde Coletiva Salvador BA Brasil Universidade Federal da Bahia. Instituto de Saúde Coletiva. Programa de Pós-Graduação em Saúde Coletiva. Salvador, BA, Brasil

**Keywords:** Chemical Contamination, Agrochemicals, Breast Milk, Exposure, Human Health

## Abstract

**OBJECTIVE:**

To identify the prevalence of contamination by pesticides and their metabolites in the milk of lactating mothers in Latin America.

**METHODS:**

In this systematic review, the PubMed, LILACS, Embase, and Scopus databases were searched up to January 2022 to identify observational studies. The Mendeley software was used to manage these references. The risk of bias assessment was evaluated according to the checklist for prevalence studies and writing design, by the Prisma guidelines.

**RESULTS:**

This study retrieved 1835 references and analyzed 49 studies. 69.38% of the analyzed studies found a 100% prevalence of breast milk contamination by pesticides among their sample. Main pesticides include dichlorodiphenyltrichloroethane (DDT) and its isomers (75.51%), followed by the metabolite dichlorodiphenyldichloroethylene (DDE) (69.38%) and hexachlorocyclohexane (HCH) (46.93%). This study categorized most (65.30%) studies as having a low risk of bias.

**CONCLUSIONS:**

This review shows a high prevalence of pesticide contamination in the breast milk of Latin American women. Further investigations should be carried out to assess contamination levels in breast milk and the possible effects of these substances on maternal and child health.

## INTRODUCTION

The World Health Organization (WHO) recommends exclusive breastfeeding (EBF) for infants up to six months of life and, after that period, complementing it with other foods up to two years of life or more^1,2^. Scientific evidence shows the protective effects of breast milk for infants’ health as it contains energy; essential nutrients for their nutritional, metabolic, and physiological demands up to the sixth month of life; and immunological and cellular components^3^ that protect children against diarrhea, respiratory infections, and infant morbi-mortality^4^.

Despite breast milk being considered an ideal food for child nutrition and development, its composition and quality can change due to external environmental factors, such as mothers’ diet and lifestyle^5^.

Substances in pharmaceuticals, tobacco, alcohol, illicit drugs, and chemical products (such as pesticides) can be secreted into breast milk^6,7^, interfering with the quality of this food regarding its composition, nutritional value, and aroma, hindering the milk ejection reflex and infants’ suction, which contributes to reducing breastfeeding time^8^. For these reasons, exposure to such substances should be discouraged, especially during pregnancy and breastfeeding^6^.

Pesticides constitute chemical, physical, or biological products used to directly or indirectly control, destroy, or prevent pathogenic agents in plants and useful animals and people^9^. They serve as herbicides, fungicides, pesticides, rodenticides, nematicides, acaricides, molluscicides, termiticides, growth regulators and inhibitors, fumigants, fertilizers, wood preservatives, and some veterinary products^10,11^. The most commonly used compounds include organophosphates, carbamates, and halogenates, among others^10^. These substances can contaminate breast milk if in direct contact with agricultural use^12^, or indirectly, via exposure to its residues in the air, water, and animal, and vegetable foods^7^.

Some of their chemical properties give pesticides the capacity to disperse and accumulate in the environment. Moreover, those with liposoluble properties may be present in foods of animal origin, indirectly contaminating and accumulating in the adipose tissue of human beings^12^. Breast milk, in turn, has a high concentration of fats in its composition, making it a potential vehicle for transferring pesticide residues^13^.

Studies conducted in Latin America since the 1990s show the presence of pesticides in the milk of women in that region. A study conducted^14^ in Ribeirão Preto, São Paulo (Brazil) found residues of four types of pesticides, with dichlorodiphenyltrichloroethane (DDT) being identified in 100% of the analyzed breast milk samples, with higher mean levels of concentration in samples from occupationally exposed women than in those from non-exposed women. A study with women in Mexico^15^ found the presence of two compounds derived from pesticides in 76% of the analyzed milk samples.

The high prevalence of pesticide in breast milk is alarming due to the negative repercussions of pesticide to mothers’ and infants’ health. Amidst the wide range of chemical substances available in the market, estimates suggest that more than a 1000 can disrupt the endocrine system. Scientific evidence shows that pesticide exposure can lead to hormonal disorders and affect reproduction, contributing to the early onset of puberty, longer menstrual cycles, and anticipation of menopause^16^. Furthermore, it is associated with the development of breast cancer; congenital disabilities; and impaired social, cognitive, and psychomotor development^9,17^.

The importance of breast milk in infant nutrition and health the exposure of women in Latin America to chemical substances that can interfere with milk quality entail the need to systematize the scientific production in Latin America to identify the prevalence of contamination by pesticides and their metabolites in the breast milk of women directly or indirectly exposed to these compounds.

## METHODS

### Registration and Guiding Question

This is a systematic review (SR) of the literature aimed at finding studies developed in Latin America that evaluate the prevalence of pesticides and their metabolites in breast milk. This study followed the Prisma 2020 guidelines^18^ and its protocol was registered in the Prospective Register of Systematic Reviews (PROSPERO) under number CRD42018106637.

### Eligibility Criteria

Studies were selected following the PECOT acronym: P (Population) - studies with lactating mothers in Latin America; E (Exposure) - Human breast milk; C (Comparison) - no comparisons were made between groups in this systematic review; O (Outcome) - contamination of breast milk by pesticides; T (Study Type) - original articles, theses, and dissertations with observational study designs (cohort, cross-sectional, and case-control studies).

### Search Strategy

The high sensitivity data search was independently carried out by two researchers on the PubMed, LILACS, Embase, and Scopus databases using the following descriptors: “breast feeding”, “breast milk”, “agrochemicals”, “pesticides”, “contamination”, and their respective terms in Portuguese and Spanish, which were combined with sensitive Boolean operators suited to each search platform (as described in Table 1).

**Table 1:** Search strategies in databases and main results, 2022.

Database	Search strategy	Items found
PubMed/Medline	#1 “Milk, Human”[Mesh] OR (Breast Milk) OR (Milk, Breast) OR (Human Milk) #2 “Pesticides”[Mesh] #3 “Pesticide Residues”[Mesh] OR (Pesticide Residue) OR (Residue*, Pesticide) #4 “Agrochemicals”[Mesh] OR Agrichemical* OR (Agricultural Chemical*) OR (Chemical*, Agricultural) OR Agrichemical* #5 “Insecticides”[Mesh] #6 #2 OR #3 OR #4 #5 #1 AND 6	829
Embase	#1 ‘breast milk’ OR (breast fed infant) OR (homogenized pasteurized human milk) OR (human milk) OR (maternal milk) OR (milk, human) OR (milk, mother) OR (mother milk) OR (woman milk) #2 ‘pesticide’ OR (agent, pesticide) OR (pesticidal agent) OR (pesticide agent) OR (pesticide synergists) OR pesticides #3 ‘pesticide residue’ OR (residue, pesticide) #4 ‘organophosphate pesticide’ OR (organic phosphate pesticide) OR (organophosphorus pesticide) OR (pesticide, organophosphoric) OR (phosphoroorganic pesticide) #5 ‘insecticide’ OR (insecticidal agent) OR (insecticide agent) OR (insecticides, botanical) #6 #2 OR #3 OR #4 OR #5 #6 #1 AND #6	120
Scopus	#1 ( “breast milk” ) OR ( “breast fed infant” ) OR ( “homogenized pasteurized human milk” ) OR ( “human milk” ) OR ( “maternal milk” ) OR ( “milk, human” ) OR ( “milk, mother” ) OR ( “mother milk” ) OR ( “woman milk” ) #2( “insecticides” ) #3 ( “pesticide residues” OR “pesticide residue” OR “residue*, pesticide” ) #4 ( “pesticides” ) #5 #2 OR #3 OR #4 #6 #1 AND #5	862
Lilacs	#1 MH: “Leite Humano” OR (Milk, Human) OR (Leche Humana) OR (Leite Materno) OR MH:A12.200.467$ OR MH:A12.790.500$ OR MH:G07.203.100.700.500$ OR MH:G07.203.300.350.525.500$ OR MH:J02.200.700.500$ OR MH:J02.500.350.525.500$ OR MH:SP6.021.057.073.159$ #2 MH:”Resíduos de Praguicidas” OR (Pesticide Residues) OR (Residuos de Plaguicidas) OR MH:D27.720.031.700.672$ OR MH:D27.888.723.697$ OR MH:N06.850.460.200.700$ #3 MH:”Agroquímicos” OR Agrochemicals OR Agroquímicos OR Agrotóxico* OR (Defensivo Agrícola*) OR (Produto Agroquímico*) OR (Produto Químico Agrícola) OR (Substância Agroquímica*) OR (Substância Química Agrícola*) OR MH: D27.888.723.697$ #4 #2 OR #3 #5 #1 AND #4	24

### Studies Selection

The Mendeley Desktop software (version 1803) was employed to manage the retrieved references, remove duplicates, and apply the inclusion criteria to the chosen studies. The titles were first read; then, the abstracts, and finally the whole manuscripts.

### Risk of Bias

The 2017 Joanna Briggs Institute (JBI) Checklist for Prevalence Studies^19^ was used to assess the rigor and methodological quality of all 49 studies chosen for this review. This instrument is composed of nine questions, including adequacy of the sample structure, recruitment of the population, adequacy of sample size, detailing the subjects and study setting, adequacy of data analysis, employment of validated methods, criteria for measuring variables, and adequacy of response rates.

Positive responses to any of the nine questions that compose the checklist were considered to classify risk of bias. Studies that had up to a 49% “yes” score were categorized as high risk of bias; those with a positive response for 50%-69% of the questions, as moderate risk; and studies that had a positive score for 70% or more of the questions, as low risk^19^.

### Data Extraction and Analysis

Data were extracted by a reviewer and checked by another with the help of a standardized spreadsheet on Microsoft Excel containing the following variables: names of the authors, title of the article, year of publication, region of the study, country of origin, sample, age and income of participants, presence of pesticide contamination in breast milk, methodology used to identify pesticides in breast milk, types of pesticides and metabolites found in breast milk, and number of women exposed and not exposed to pesticides.

The prevalence of pesticide in breast milk was calculated according to the following formula: total number of breast milk samples contaminated by pesticides divided by the total number of samples multiplied by 100. Results were organized in graphs and tables.

## RESULTS

### Search Results

**Figure 1:**
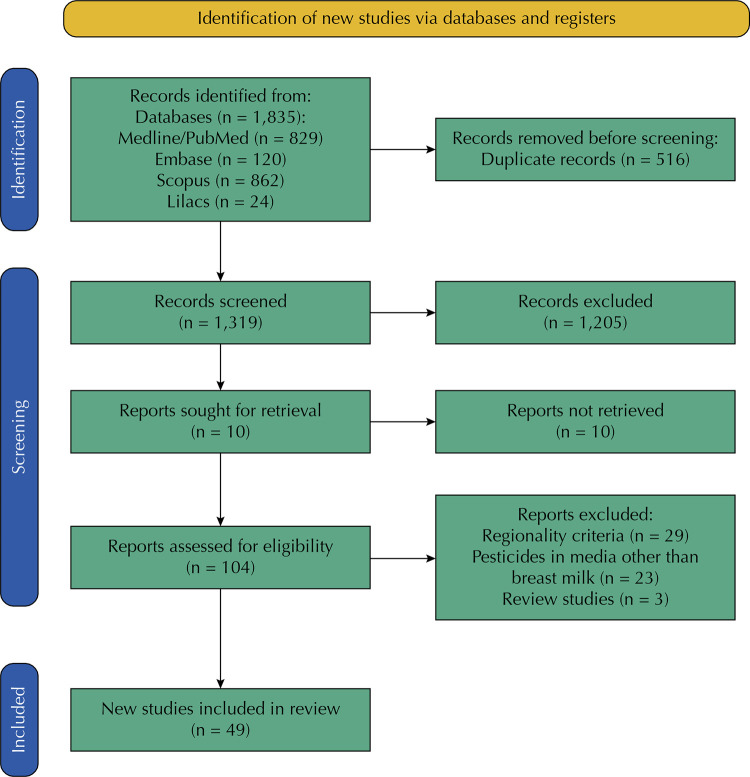
Flowchart of the search strategy used in this systematic review.

A total of 1835 references were identified. After reading their titles and abstracts and applying the eligibility criteria, 104 articles were selected to be read in full, of which 49 articles^14,15,20-67^ were selected for study (Figure 1). The main reasons for excluding articles referred to their failure to meet the established regionality criteria (n = 29), research of pesticides in media other than breast milk (n = 23), and review studies (n = 3).

### Characteristics of the Eligible Studies

All the studies included in this review were published from 1973 to 2020, most of which were from 1988 to 1999 (32.65%), conducted in Mexico (44.89%) and in Brazil (20.4%), with a cross-sectional design (79.59%), and a sample size smaller than 100 (65.3%). Most samples were collected from 1993 to 2002 (24.48%) and contemplated a rural and urban zone population (30.61%). Study participants’ age ranged from 13 to 44 years (Table 2).

**Table 2:** Characteristics of the studies included in this systematic review, 2022.

Variable	n	%
Year of publication		
1973–1987	8	16.32
1988–1999	16	32.65
2000–2011	12	24.48
2012–2020	13	26.53
Data collection		
1970–1981	5	10.2
1982–1992	11	22.44
1993–2002	12	24.48
2003–2014	6	12.24
2015–2020	1	2.04
Country		
Not informed	14	28.57
Mexico	23	46.93
Brazil	10	20.4
Nicaragua	4	8.16
Guatemala	3	6.12
Chile	2	4.08
Colombia	2	4.08
Costa Rica	1	2.04
Spain	1	2.04
French West Indies	1	2.04
Panama	1	2.04
Venezuela	1	2.04
Sample size		
< 100	32	65.3
101–191	12	24.48
192–300	5	10.2
Study type		
Cross-sectional	39	79.59
Cohort	10	20.40
Rural and urban	15	30.61
Urban only	3	6.12
Rural only	3	6.12
Not informed	28	57.14

### Risk of Bias Results

**Figure 2:**
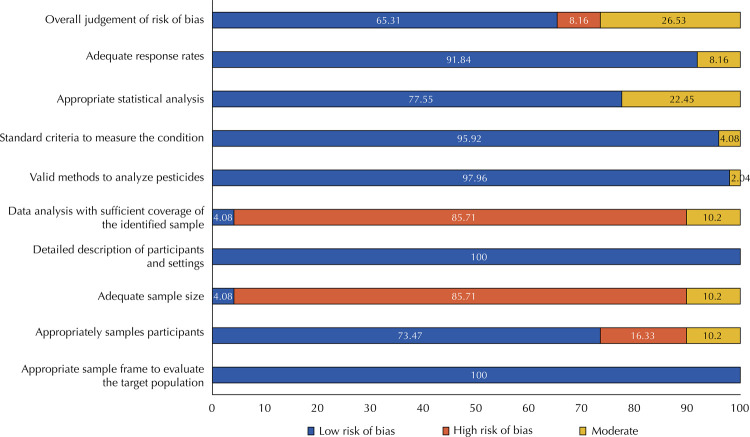
Risk of bias of the studies included in this systematic review using the Instituto Joanna Briggs critical appraisal tool for prevalence studies.

This review individually assessed risk of bias by the JBI Checklist for Prevalence Studies. After the methodological assessment following the JBI criteria, this systematic review included all 49 studies, categorizing most (65.30%) of them as having a low risk of bias; 26.53%, as with a medium risk of bias; and 8.16%, as with a high risk of bias (Figure 2). The criteria that scored most negatively among the selected studies referred to sample size adequacy (85.71%), analysis with sufficient sample coverage (85.71%), and recruitment of study participants (16.32%).

### Prevalence and Main Pesticides Found in the Breast Milk of Women in Latin America

This review found pesticide contamination in the milk of lactating mothers in all included studies and a prevalence of pesticides ranging from < 30% (n = 1), 70 to 99.9% (n = 10), and 100% (n = 34), with the most frequent pesticides including dichlorodiphenyltrichloroethane (DDT) (75.51%), followed by dichlorodiphenyldichloroethylene (DDE) (69.38%) and hexachlorocyclohexane (HCH) (46.93%) (Table 3).

**Table 3:** Prevalence and types of pesticide contamination of lactating mothers’ milk in Latin America in the studies included in this systematic review, 2022.

Variable	n	%	References
Types of pesticides			
DDT (o.p’-DDT, p.p’-DDT)	37	75.51	^14,15,28–37,20,38–47,21,48,52,63–67,22–27^
DDE (p.p’-DDE, o.p’-DDE)	33	67.34	^20,21,32–39,41,44,22,45–47,49–55,24,64,65,67,25–29,31^
HCH (alpha HCH, γ-HCH, β-HCH, s-HCH)	23	46.93	^14,15,42,43,46–48,51,52,54–56, 20,64,65,67,25,28,29,35,38,40,41^
Dieldrin	13	26.53	^14,24,56,57,66,27,32,36,40,47,48,54,55^
DDD (p.p’-DDD)	12	24.48	^21,24,52,64,26–29,32,37,44,47^
Benzyl chlorides^a^	11	22.44	^15,20,67,28,29,35,40,47,52,55,65^
Heptachlor	7	14.28	^14,27,47,48,51,55,64^
Heptachlor epoxide	7	14.28	^24,32,35,36,40,55,66^
Endrin (aldehyde)	5	10.20	^24,32,39,47,66^
Aldrin	5	10.20	^24,39,47,54,66^
Endosulfan (II)	2	4.08	^57,66^
Chlordane (β-chlordane, γ-chlordane)	2	4.08	^51,54^
Organochlorines^b^	5	10.20	^27,47,55,57,58^
Pyrethroids	2	4.08	^37,59^
Not identified	1	2.04	^60^
Chlordecone	1	2.04	^61^
PCBs	1	2.04	^62^
Prevalence of pesticides in breast milk			
< 30%	1	2.04	^62^
30 to 69%	2	4.08	^48,55^
70% to 99.9%	10	20.4	^15,21,29,42,47,49,51,61,64,66^
100%	34	69.38	^14,20,32–41,22,43–46,50,52–54,57,58,24,59,63,65,67, 25–28,30,31^
Not identified	2	4.08	^23,60^

DDT: dichlorodiphenyltrichloroethane; DDE: dichlorodiphenyldichloroethylene; HCH: hexachçorocyclohexane; DDD: dichlorodiphenyldichloroethane; PCBs: polychlorinated biphenyls.

^a^ Hexachlorobenzene, tetrachlorobenzene, pentachlorobenzene.

^b^ Methoxychlor, mirex, and toxaphene.

## DISCUSSION

This systematic review found a high prevalence of pesticide contamination in women’s breast milk in 11 Latin American countries from 1973 to 2020, of which 69.38% of the included studies found a 100% prevalence of pesticide contamination of breast milk among their sample. Of these, de Campos and Olszyna-Marzys^40^ (carried out in Guatemala and El Salvador) stands out for its alarming data, in which, with the exception of one sample, all others showed pesticide contamination levels above the adequate limits for human consumption.

This high prevalence of pesticide contamination may be related to the unrestricted use of these substances in agricultural production since Latin American countries produce commodities for the international market^68^. Among them, Brazil stands out as the biggest consumer of agrochemicals in the world since 2008^68^ and as the second biggest buyer of substances already banned in Europe^69^. Moreover, the number of pesticides released in the country has increased since 2015, reaching the highest rate of pesticide release in a year in 2019^70^.

In this systematic review, Brazil had the second highest production of data in relation to the presence of pesticides in breast milk, with most studies finding a high prevalence of contamination^14,42,43,45-48,55,57^. The study by Souza et al.^57^(carried out in western Bahia, Brazil) stands out as they found two or more pesticide residues in all 34 analyzed samples, with methoxychlor, dieldrin, and endosulfan in highest mean concentrations.

The flexibilization of the legislation that regulates the use and sale of agrochemicals in Brazil may be reflected in the data from the analyzed studies since the prevalence of breast milk contamination varied from 65% to 100%.

On the other hand, Mexico has produced the most data on the presence of pesticides in breast milk, with a prevalence of contamination ranging from 76% to 100%. The pesticides registered and authorized for use in the country by its Federal Commission for Protection against Sanitary Risks in 2016 include 140 active ingredients that other countries have banned or unauthorized. Of these, 65 pesticides are considered highly dangerous according to the criteria established by the FAO and WHO^71^.

Although the workers that directly handle these products (whether in transport/commerce, formulation, or application in farming and livestock) suffer more intense exposure, the general population also finds itself susceptible to contact with these chemical substances, primarily by food via the intake of residues in both unprocessed and processed foods^72^. This occurs because these substances not only persist in the environment for prolonged periods, but most also have a bioaccumulation capacity, contributing to the contamination of the soil, water, and foods^12^.

Unprocessed and minimally processed foods, the basis for a nutritious diet, are recommended for mothers who are in the phase of producing breast milk and have a greater demand for energy and micronutrients^73^. However, studies show that pesticide residues are increasingly contaminating these foods.

In all the years analyzed, data from the reports of the Program for Analyzing Pesticide Residues in Foods from 2008 to 2015 recorded pesticide residues above the maximum permitted residue limit or residues of unauthorized pesticides in pepper and cucumber crops, whereas more than half of pepper crop samples showed irregularities in all years^74^.

Other studies also show pesticide contamination in foods frequently consumed by the Brazilian population, such as those conducted by Lemes et al^75^, which found pesticide residues in 26 (59%) rice samples and in 11 (25%) bean samples, and Silva et al.^76^, which found pesticide residues in Fuji- and Argentina-type apples sold in the Mooca region in the municipality of São Paulo.

Such chemical substances also widely contaminate processed foods. A study conducted by the Brazilian Consumer Defense Institute found at least one type of pesticide in 16 (59.3%) of the 27 analyzed ultra-processed foods, of which all those that contained wheat in their composition included pesticide residues^77^. It warrants highlighting that products derived from wheat are well accepted and widespread in the diet of Latin American populations.

Some studies in this review found higher levels of pesticides in women’s breast milk in rural and suburban areas, which may be associated with occupational exposure on large farms. Large-scale food production can create farmer dependency on agrochemicals for cultivation^15,22,25,26,31,42,48,52,59^. However, other studies^42,44^ found higher levels of DDT in breast milk samples derived from women in the urban zone. These findings are associated with the dietary habits of that population, which show higher meat consumption than rural populations^42^.

Amidst the intense use of pesticides for food production, organic farming offers an excellent alternative to traditional means of production. By adopting sustainable practices without the use of pesticides and chemical inputs, it contributes not only to protecting the health and healthy development of future generations, but also to preserving biodiversity and the environment^78^. This agricultural approach based on agroecological principles reduces the risks associated with pesticide contamination and promotes the preservation of local ecosystems.

It is also important to place greater value on food production derived from small farmers that use alternative inputs instead of the conventional methods adopted by agribusiness (pesticides, transgenic seeds, and chemical fertilizers)^79^.

Notably, the Dietary Guide for the Brazilian population^80^, for children aged under two years^81^, and the Mexican^82^ and Guatemalan^83^ dietary guides recommend that unprocessed and minimally processed foods should constitute the basis for an adequate and healthy diet and that the consumption of processed and ultra-processed foods should be limited. These recommendations, adapted to regional dietary habits, contribute to making appropriate food choices and strengthening more sustainable production systems^81^.

Regarding pesticides and their metabolites, most studies (75.51%) found DDT and its isomers, followed by its metabolite DDE (69.38%) and HCH (46.93%). Contamination by DDT has a greater relation with its inhalation and food contamination given its high topical toxicity^84^, representing more recent exposure to the substance. In turn, the metabolite DDE represents prior exposure to DDT and its persistence in the environment^85^.

HCH exposure, in turn, more frequently occurs by inhalation due to occupational use or via the digestive tract from the consumption of contaminated foods by people not occupationally exposed, most often affecting liver functions. Also, despite a lack of evidence of its carcinogenicity in human beings, experimental studies show positive findings for malignant neoplasias^86^.

Exposure to these toxic substances negatively affects the population’s health, especially more vulnerable groups such as pregnant women, infants, and breastfeeding children. Various studies relate pesticide exposure with adverse effects on leukocyte growth and development, thus interfering with bodily immune functions^87^, causing dysfunctions to the nervous, reproductive, and endocrine system^12^ and increasing the risk of developing breast, digestive, genital, urinary, and respiratory cancer^88^.

Considering the different possibilities of pesticide exposure, it is plausible to infer that breast milk contamination could be substantially higher than currently estimated, especially if we consider that most existing studies tend to focus on a limited set of pesticides, which may underestimate the reality of exposure. Therefore, more comprehensive research is essential to identify and quantify these chemical compounds, providing a more accurate view of potential risks to maternal and child health.

Breast milk is the ideal food for child nutrition and development, especially in the first months of children’s lives, and it should not contain undesirable chemical substances. Pesticides and the health problems related to their use configure a serious public health problem because child and breastfeeding infant exposure to pesticides may be associated with various health problems, such as the development of leukemia and other types of cancer, low birth weight, birth defects, cognitive deficit, and a low intelligence quotient^89^.

Results of a longitudinal study suggest that fetal exposure to DDE during the first trimester of pregnancy can negatively affect children’s psychomotor development during their first year of life^90^. Several studies have also shown an association between fathers who were exposed to pesticides and congenital malformations in their children, especially those related to the male reproductive system^91^, central nervous system outcomes^92^, and fetal growth restrictions^93^.

In women, this exposure is associated with endocrine/hormonal dysregulation, breast cancer, risk of depression^9^, kidney diseases, predisposition to ovarian and thyroid cancer, diabetes, and an increased likelihood of polycystic ovary syndrome^94^.

Although beyond the scope of this study, some factors worsen the toxic effects of pesticides, such as malnutrition — 53.7 million people lived in poverty and under severe food insecurity in Latin America from 2016 and 2018^89^. These numbers tend to increase given the COVID-19 pandemic and the related political and economic instability that resumed food and nutritional insecurity—with a significant expression of hunger in Latin American countries—and should thus be considered in this discussion^95^.

### Strengths and Limitations

Some limitations of this systematic review are related to the relative need for up-to-date studies in Latin America. Most of the available studies that show the levels of contamination of breast milk in these countries are old. So, more studies must be carried out to identify current contamination levels, covering sample numbers that represent the studied population since the risk of bias analysis in this study showed inadequate sample sizes that may raise the risk of bias in the sample of this review. Therefore, we ignored a meta-analysis with the data from these studies.

Nonetheless, we believe that there is no interference in the results of this review since it involves prevalence studies without an associative purpose; includes the methodological rigor employed by its independent reviewers; and involves the databases recommended by systematic review guidelines, gray literature, and the assessment of the risk of bias in the studies that met its eligibility criteria, rendering a consistent study that produced useful results for public health.

### Recommendations

The systematization of scientific production in Latin America showed a high prevalence of pesticides and their metabolites in the breast milk of women in that region, suggesting that direct or indirect exposure to pesticides configures a condition in these women’s everyday lives that can be considered a serious public health problem in the studied group given its negative impact on maternal and infant health. We should highlight that these results are unable to support the interruption of exclusive breastfeeding since contamination reflects environmental and occupational exposure and the consumption of foods containing pesticide residues.

Results may contribute to broadening the knowledge on the topic and educating the population about the violation of rights associated with the indiscriminate release of pesticides in the studied countries. However, additional epidemiological investigations with adequate sample sizes are recommended for the evaluation of breast milk contamination levels and the identification of the possible potentiators of the effects of these metabolites on maternal and infant health over time.

Moreover, public health interventions are needed for the pre-natal and post-partum period to guide women toward strategies to prevent and minimize pesticide exposure and guarantee the stimulation of exclusive breastfeeding up to the sixth month of infants’ lives (complemented up to two years or more) to ensure children’s healthy growth and development.
